# Raman-Deuterium Isotope Probing and Metagenomics Reveal the Drought Tolerance of the Soil Microbiome and Its Promotion of Plant Growth

**DOI:** 10.1128/msystems.01249-21

**Published:** 2022-02-01

**Authors:** Jee Hyun No, Susmita Das Nishu, Jin-Kyung Hong, Eun Sun Lyou, Min Sung Kim, Gui Nam Wee, Tae Kwon Lee

**Affiliations:** a Department of Environmental Engineering, Yonsei University, Wonju, Republic of Korea; University of California, Davis

**Keywords:** drought, drought tolerance, Raman-DIP, phenotype, metagenomics, soil microbiome

## Abstract

Drought has become a major agricultural threat leading crop yield loss. Although a few species of rhizobacteria have the ability to promote plant growth under drought, the drought tolerance of the soil microbiome and its relationship with the promotion of plant growth under drought are scarcely studied. This study aimed to develop a novel approach for assessing drought tolerance in agricultural land by quantitatively measuring microbial phenotypes using stable isotopes and Raman spectroscopy. Raman spectroscopy with deuterium isotope probing was used to identify the Raman signatures of drought effects from drought-tolerant bacteria. Counting drought-tolerant cells by applying these phenotypic properties to agricultural samples revealed that 0% to 52.2% of all measured single cells had drought-tolerant properties, depending on the soil sample. The proportions of drought-tolerant cells in each soil type showed similar tendencies to the numbers of revived pea plants cultivated under drought. The phenotype of the soil microbiome and plant behavior under drought conditions therefore appeared to be highly related. Studying metagenomics suggested that there was a reliable link between the phenotype and genotype of the soil microbiome that could explain mechanisms that promote plant growth in drought. In particular, the proportion of drought-tolerant cells was highly correlated with genes encoding phytohormone production, including tryptophan synthase and isopentenyl-diphosphate delta-isomerase; these enzymes are known to alleviate drought stress. Raman spectroscopy with deuterium isotope probing shows high potential as an alternative technology for quantitatively assessing drought tolerance through phenotypic analysis of the soil microbiome.

**IMPORTANCE** Soil microbiome has played a critical role in the plant survival during drought. However, the drought tolerance of soil microbiome and its ability to promote plant growth under drought is still scarcely studied. In this study, we identified the Raman signature (i.e., phenotype) of drought effects from drought-tolerant bacteria in agricultural soil samples using Raman-deuterium isotope probing (Raman-DIP). Moreover, the number of drought-tolerant cells measured by Raman-DIP was highly related to the survival rate of plant cultivation under drought and the abundance of genes encoding phytohormone production alleviating drought stress in plant. These results suggest Raman-DIP is a promising technology for measuring drought tolerance of soil microbiome. This result give us important insight into further studies of a reliable link between phenotype and genotype of soil microbiome for future plant-bacteria interaction research.

## INTRODUCTION

Drought is a major agricultural threat that limits the productivity of crops. Climate change is leading to drought periods becoming longer and more intense, due to changes in global air temperature and precipitation patterns (1). Drought stress has serious effects on the regulation of phytohormones, which play major roles in the proper phenotypic responses of plants at the physiological level. Drought can change plant phenotypes as well as symbiotic microbiomes, either directly or indirectly. The reduction of soil moisture and the restriction of access to nutrients can both directly affect soil microbiome diversity and compositional changes (2). These influences also affect the soil microbiome; they can change the composition and volume of root exudates containing sugars, amino acids, and phytohormones, which are altered by stress-induced phenotypes (3). The drought-adaptable species of the soil microbiome tend to dominate or show increased activity by using survival strategies such as osmolyte accumulation, biofilm formation, and antioxidant molecule synthesis (4–6). Some of these bacteria can promote plant growth in drought by sharing their survival strategies during drought, or by producing phytohormones or their precursors (7, 8). For instance, 1-aminocyclopropane-1-carboxylate (ACC) deaminase and indole-3-acetic acid (IAA), which are produced by drought-tolerant bacteria (DTB), reduce the ethylene levels produced by plants due to drought stress, thus preventing the deterioration of plant growth (9). DTB play a major role in the soil microbiome by maintaining crop productivity.

Despite the importance of DTB in the soil microbiome, it is challenging to study the association between the metabolic activity of DTB and plant drought tolerance. Most previous studies have experimentally evaluated the activity of DTB regarding plant growth by injecting DTB into the roots of plants under drought (9). While this culture-based approach is suitable for studying symbiosis between a single bacterial species and a plant, its ability to systematically evaluate the drought tolerance of a highly diverse soil microbiome is limited. Metagenomic approaches have confirmed that the abundances of various strains belonging to classes *Alpha-*, *Beta-*, and *Gammaproteobacteria*, *Actinobacteria*, *Bacilli*, and *Planctomycetia* increase rapidly under drought stress (2, 10, 11). In addition to the drought survival strategies mentioned above, expression levels of genes associated with various metabolic processes such as osmolyte accumulation, biofilm formation, antioxidant molecule synthesis, and phytohormone synthesis have also been demonstrated to increase in DTB (12). These results suggest that there is a high diversity of bacteria with drought-tolerant capabilities in a variety of metabolic pathways within the soil microbiome. Although metagenomic approaches can obtain overall information from soil microbiomes under drought conditions, the experimental preparation and analysis processes are costly, computationally intensive and time-consuming, limiting their application in the field. Without overlooking the importance of studying the presence and activity of DTB in the soil, there is a need for a simple quantitative assessment of the drought tolerance of the soil microbiome.

Raman spectroscopy is an emerging technology for identifying bacterial phenotypes through measuring biomolecular structures (e.g., protein, lipid, amino acid, and carbohydrate) in a cell (13); this is often referred to as a bacterial fingerprint. Due to its low-cost, label-free, and non-destructive features, Raman spectroscopy has been widely applied in bacterial detection and physiology (14, 15). Single cell Raman spectra (SCRS) can also provide phenotypic information regarding single cells, including changes in response to environmental stress for antibiotics, heavy metals, and temperatures, at a high resolution (16–18). Raman spectroscopy with stable isotope probing is a direct method that can identify unculturable bacteria with metabolic activity in an environment by measuring the isotope-induced peaks in SCRS (19). SCRS information (specifically labeled ^13^C and ^15^N from environmental samples that have been incubated with ^13^CO_2_ and ^15^N_2_) can be used to distinguish carbon and nitrogen-fixing bacterial strains, respectively (20, 21). Recently, Raman spectroscopy combined with deuterium isotope probing (Raman-DIP) has been employed to identify the metabolic activities of bacterial cells in a complex microbial community by measuring the generated carbon-deuterium (C-D) band in the presence of deuterium (22). Unlike Raman-stable isotope probing, Raman-DIP is a comprehensive labeling technique that labels all bacterial cells that exhibit metabolic activity in a given condition with deuterium. In particular, previous studies have used Raman-DIP to detect or quantify stress-tolerant bacteria in environmental samples while treating abiotic stresses including antibiotics, carbon starvation, and UV light (23–25). For instance, using information on the presence or absence of the C-D band in SCRS, quantitative comparisons have been successfully made regarding the proportion of microorganisms with metabolic activity to antibiotic resistance from a number of river samples (23). Raman-DIP can be used to evaluate the intensity of metabolic activity of bacteria under different levels of stress. Thus, Raman-DIP could potentially be used to quantitatively assess bacterial cells that are tolerant to drought stress in soil.

This study aimed to evaluate the drought tolerance of the soil microbiome using Raman-DIP, and to assess its association with plant growth promotion (PGP) in drought. We hypothesized that the phenotypes of bacterial cells with drought-tolerant capabilities would be distinguishable through Raman-DIP, and that the drought-tolerant capability of soil microbiome could be quantified by counting DTB in soil. It was assumed that soil with a high DTB abundance would have many PGP-related genotypes, which would help to promote plant growth in drought. In this study, the specific Raman spectra signatures of DTB were obtained by comparing six drought-tolerant and drought-sensitive bacterial species. Using this spectral information, the drought-tolerant capability of soil was quantified by counting the DTB in agricultural soil samples. In addition, the quantified phenotypic information was cross-validated by comparing the plant phenotype and genotype of the soil microbiome through pot tests and metagenomics-based approaches, respectively. This study explored the ability of Raman-DIP to quantitatively assess the drought tolerance of the soil microbiome.

## RESULTS

### Bacterial growth under drought stress.

Comparing the growth of bacterial cells under drought stress to those under normal conditions (Fig. S1) reveals that polyethylene glycol (PEG) treatment inhibited the growth of drought-sensitive bacteria (DSB), but that DTB was able to grow under drought stress although the growth rates decreased following PEG treatment. The tolerant strains exhibited significantly differing growth inhibition levels to those under normal conditions (Wilcoxon test, *P* < 0.05). These results confirmed that the incubation conditions of the 25% PEG treatment were sufficient for evaluating drought-tolerance.

10.1128/msystems.01249-21.1 FIG S1 Growth curves for DTB and DSB. Growth curves in liquid phase were obtained using optical density measurements at 600 nm. Values shown with SD were obtained from independently triplicate-measured values. Bacteria were incubated for 24 h in media (control) with PEG (PEG treatment). Download FIG S1, TIF file, 1.1 MB. Copyright © 2022 No et al. 2022 No et al. https://creativecommons.org/licenses/by/4.0/ This content is distributed under the terms of the Creative Commons Attribution 4.0 International license.

### Ability of Raman-DIP to evaluate drought tolerance.

**FIG 1:**
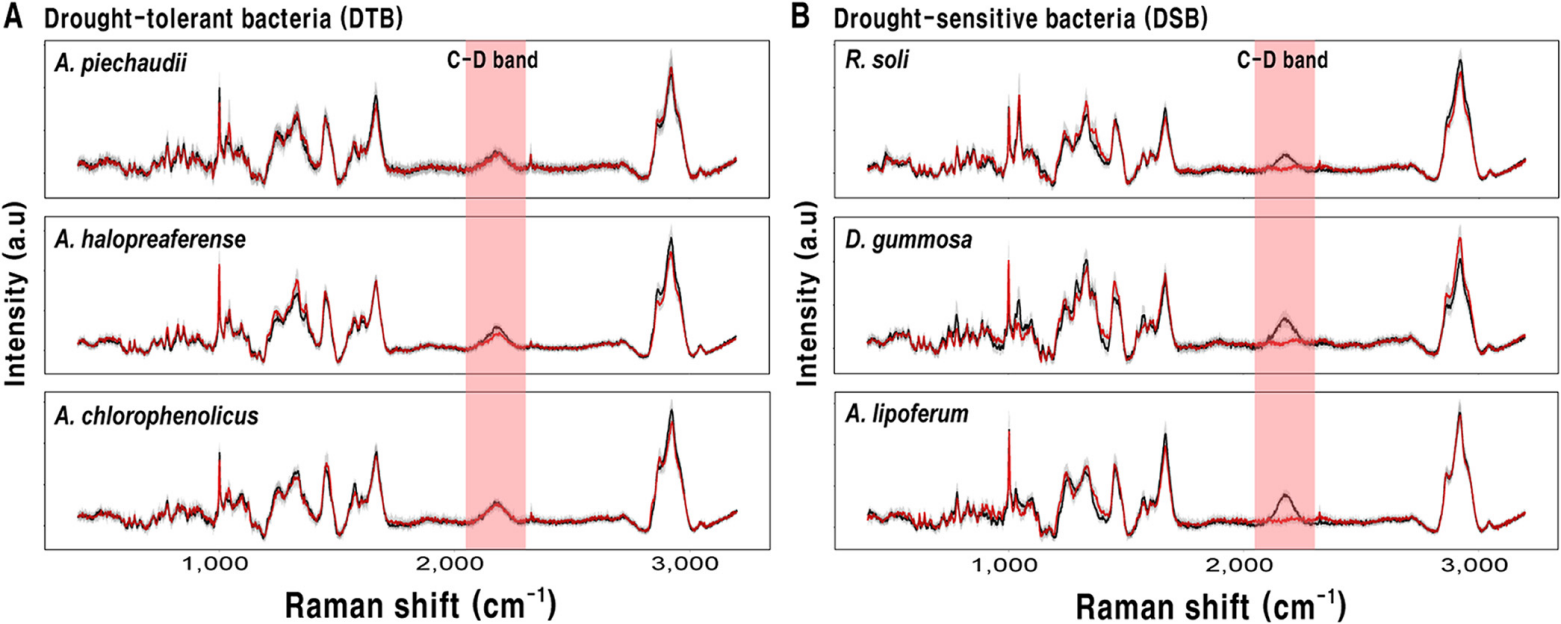
SCRS of tolerant and sensitive bacteria after incubation. SCRS are for media with either with 0% (black line) or 25% PEG (red line), including 40% deuterium water. SCRS of 20 cells were averaged (solid line) with SD shown in gray shading. (A) DTB: *A. piechaudii*, *A. halopraeferens*, and *A. chlorophenolicus*. (B) DSB: *R. soli*, *D. gummosa*, and *A. liporerum*.

Employing the C-D band as a biomarker of microbial activity, which was a broadband Raman shift between 2,040 and 2,300 cm^−1^, allowed the differentiation of inactive or active cells by adding D_2_O (22). The C-D band was displayed in the SCRS of all tested strains grown with D_2_O under normal conditions (Fig. 1). The SCRS of DTB under PEG treatment showed a detectable C-D band (Fig. 1A), whereas the C-D band of DSB disappeared (Fig. 1B). These results support that DTB maintained microbial activities when treated with PEG.

### Raman-based phenotypic profiling of drought tolerance.

**FIG 2:**
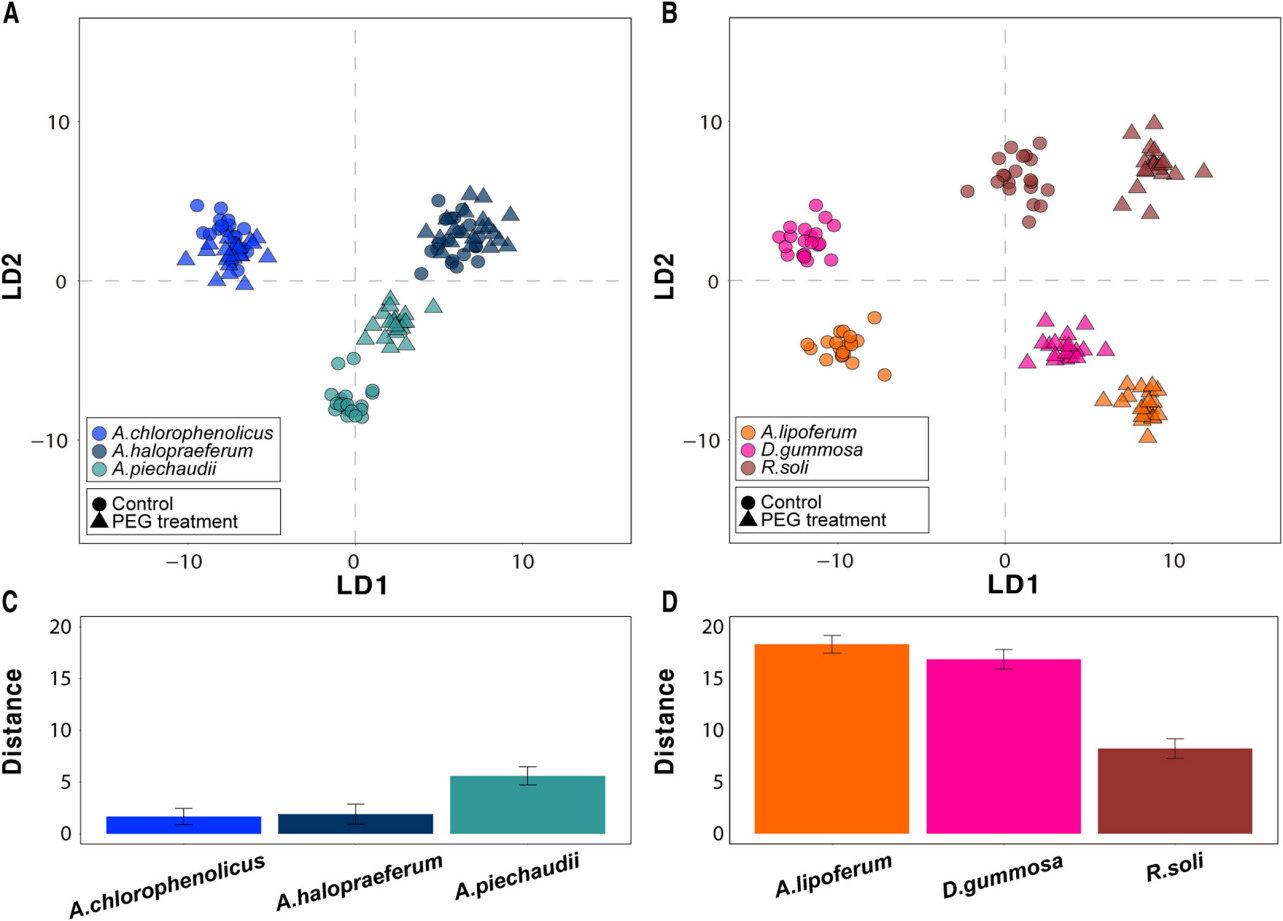
DAPC plot of SCRS. SCRS of fingerprint region for (A) DTB and (B) DSB. Distances are averages and SD between each centroid of the control group and each dot of the PEG-treated group (C and D). The six colors in the plots represent the six species. The solid circles and triangles represent the control and PEG treatments, respectively.

The SCRS of the DSB and DTB were used to analyze how cell phenotypes changed under drought stress. The discriminant analysis of principal components (DAPC) plots of DSB and DTB showed obvious differences depending on the incubation conditions (Fig. 2). The groups of the control and PEG treatments for DTB were tightly clustered (Wilcoxon test, *P* > 0.05) (Fig. 2A), whereas the clusters of DSB under PEG treatment were clearly divided from those of the control (Wilcoxon test, *P* < 0.001) (Fig. 2B). The mean of distances between each centroid of the control and PEG treatments in DTB (3.05 ± 2.00) were considerably closer than those of DSB (14.48 ± 4.55; Fig. 2C and D). Although Achromobacter piechaudii was farther away from the centroid than were the other DTB species, it was still closer than Rhizobium soli, which was the closest DSB (Fig. 2C and D). These comparisons demonstrate that Raman spectroscopy could identify the phenotypic features caused by PEG treatment to link with drought tolerance.

**FIG 3:**
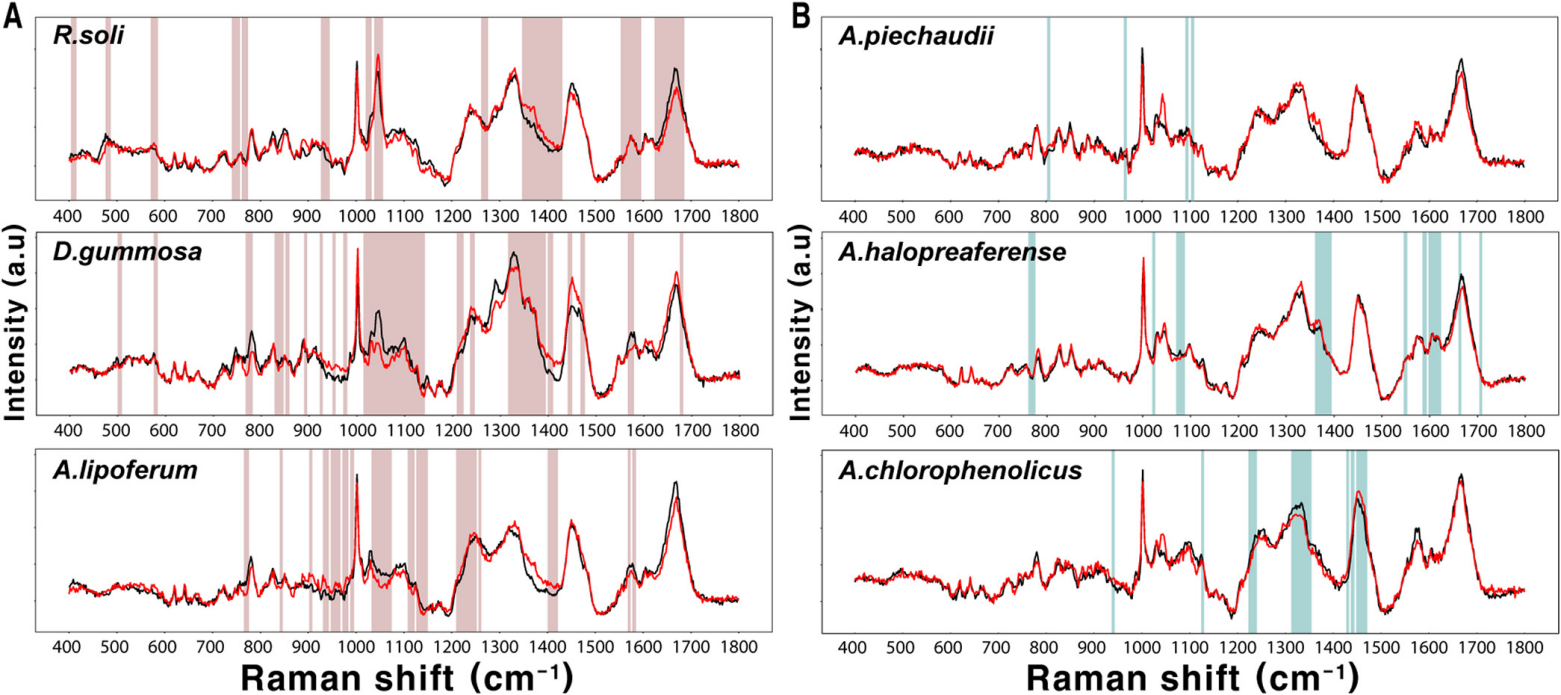
Statistical significance of Raman shifts, comparing spectra of control and PEG treatments. SCRS were measured for 20 individual cells of (A) DSB and (B) DTB under each condition. The average of SCRS is shown for control (black line) and PEG (red line) treatments. The red and blue boxes represent the Raman shifts that were significantly different between two conditions (*p* <0.01).

**FIG 4:**
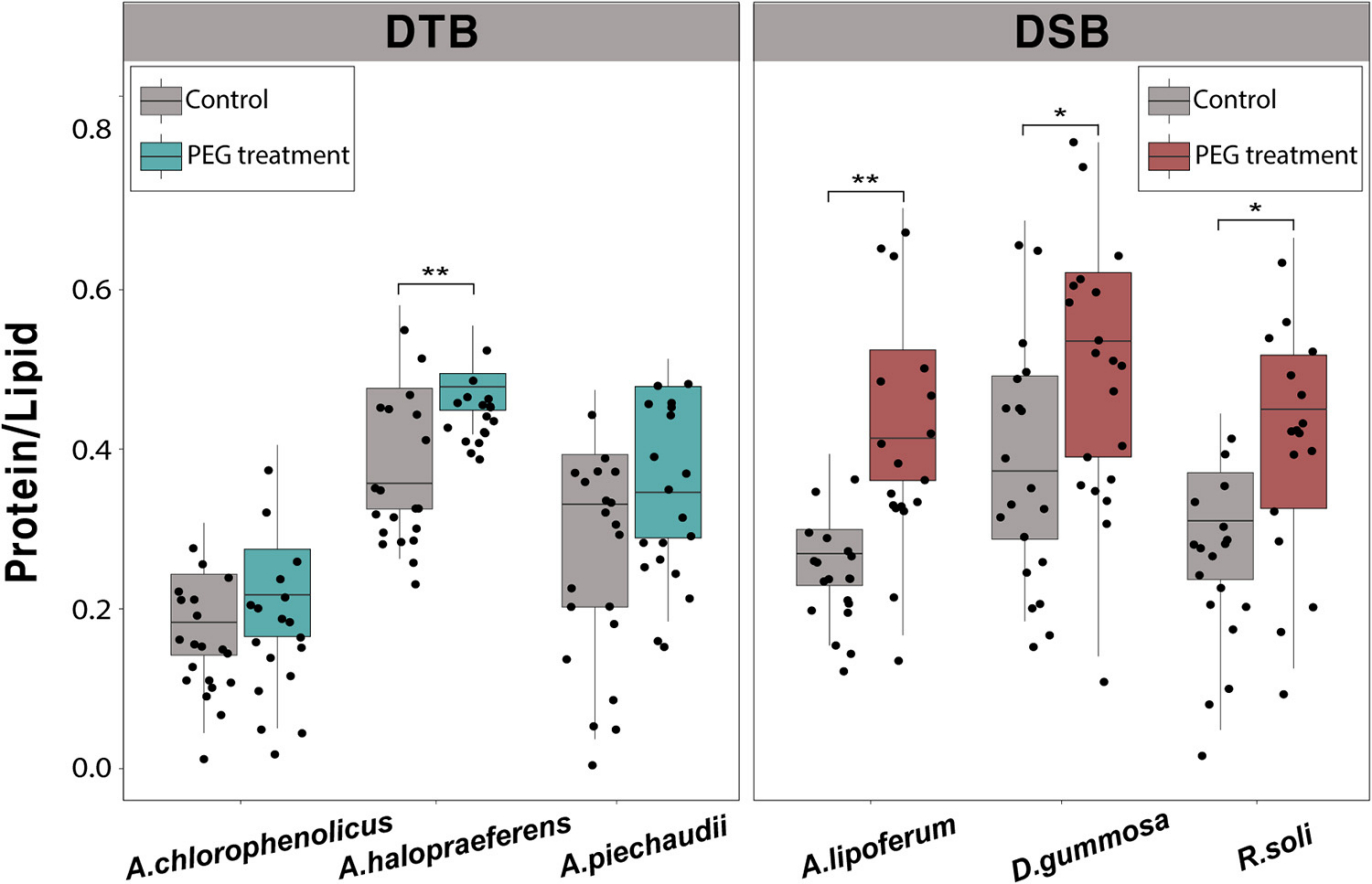
Ratio of Raman intensity for Protein/Lipid (1,209 cm^−1^/1,267 cm^−1^) with statistical analysis via *t* test. Control group was cultured in non-PEG treated media; PEG group was cultured in 25% PEG-treated media (*, *P* < 0.05; **, *P* < 0.005; and ***, *P* < 0.0005).

Phenotypic changes in the biomolecular components of bacterial cells under drought stress were analyzed to understand stress-driven changes. The PEG treatment in DTB caused very faint changes. These differences varied from species to species for DTB (Fig. 3). The Raman spectra of DSB exhibited a lot of intercellular spectral variability in the presence of PEG. Those Raman shifts which had significantly different spectral intensities in DSB and DTB when treated with PEG are summarized in Table 1 (*t* test, *p* < 0.01). Comparing the SCRS of the different strains of DSB revealed that a considerable number of peaks with intensities that were significantly altered by drought overlapped. The intensities of Raman peaks or bands corresponding to phenylalanine (620 and 1,002 cm^−1^), l-alanine (922 cm^−1^), carbohydrate and protein (936 and 1,545 cm^−1^), cholesterol (950 cm^−1^), nucleic acid (1,240, 1,375, 1,421 to 1,427, and 1,476 cm^−1^), and amide III (1,242 cm^−1^) all increased under drought stress. The intensities of Raman peaks corresponding to glucose (407 cm^−1^), aromatic compounds (1,030 cm^−1^), phenylalanine (1,032 cm^−1^), lipid (1,267 cm^−1^), nucleic acid, and amide II (1,573 cm^−1^) all decreased. The PEG treatment generally reduced the intensities of lipid-related peaks, whereas the peaks related to nucleic acid or protein appeared to increase. Ratiometric analysis was performed to compare the changes more clearly in the biomolecular compositions of bacterial cells (Fig. 4) (18). The ratio of proteins (1,209 cm^−1^) to lipids (1,267 cm^−1^) in DSB increased significantly after PEG treatment (*t* test, *p* < 0.05). In the case of DTB, excluding Azospirillum halopraeferens, there were no significant increases after PEG treatment.

**TABLE 1:** The Raman frequency with significantly different intensity in the spectra of drought-sensitive and drought-tolerant bacterial cells

Raman frequency (cm^−1^)	Assignment	Group	Ref	Raman frequency (cm^−1^)	Assignment	Group	Ref
407	Skeletal modes of carbohydrates (glucose)	Glucose	(57)	1,242	Amide III	Amide III	(58)
481	Skeletal modes of carbohydrates (starch)	Starch	(57)	1,246	Thymine, Cytosine, Adenine	Thymine, Cytosine, Adenine	(59)
620	Phenylalanine (skeletal)	Phenylalanine	(60)	1,267	Lipids	Lipids	(61)
748 to 751	O-P-O sym str	O-P-O sym str	(62)	1,270 to 1,300	Amide III (Random coils)	Amide III (Random coils)	(63)
749	Pyrrole breathing mode	Cyt c.	(20)	1,312	δ(C − H)	Cyt c.	(20)
752	δ(C–C) Tyr	Protein, Cytochrome	(64)	1,328	CH def	Peptides	(58)
782	O-P-O breathing, Cytosine, Uracil	Cytosine, Uracil	(58)	1,333	CH_3_CH_2_ def. of collagen	Nucleic acid, Protein	(16)
838	DNA	DNA	(65)	1,338	Adenine, Guanine, Tryrosine, Tryptophan	Adenine, Guanine, Tryrosine, Tryptophan	(58)
853	ν(C–C) proline, ring breath. Tyr	Protein (Glycogen, Collagen)	(64, 66)	1355	A, G, CH def.	Nucleic acid, Protein	(64)
858	CC str, COC 1,4 glycosidic link		(60)	1,375	Thymine, Adenine, Guanine	Thymine, Adenine, Guanine	(67)
922	R-CH_3_	L-alanine	(66)	1,388	CH_3_	Lipid	(16)
936	C–O–C linkage, C–C stretch., α-helix	Carbohydrate, protein	(66)	1,421 to 1,427	Adenine, Guanine	Adenine, Guanine	(67)
950	Cholesterol	Cholesterol	(16)	1,431 to 1,481	Protein marker band 1451	Protein marker band 1451	(67)
972	CH_2_ rock., C–C stretch., α-helix	Protein, Lipid	(68)	1,440 to 1,460	C-H_2_ def	C-H_2_ def	(60)
989	β-sheet	Protein, Histamine	(66)	1,441	Lipids	Lipids	(61)
1,002	Phenylalanine	Phenylalanine, b-carotene	(58)	1,445	CH_2_ scissoring	Peptides	(58)
1,030	δ(CH) bend., Tyr, Phe	Aromatic compound	(64, 66)	1,450	G, A, CH def.	Nucleic acid, Protein, Lipid, Carbohydrate	(64)
1,030 to 1,130	Carbohydrates, mainly -C-C-(skeletal), C-O, def(C-O-H)	Peptides	(57)	1,453	Protein	Protein	(59)
1,032	Phenylalanine; C-N str	Phenylalanine	(59)	1,476	Amide II, Purine bases (U)	Cytochrome, Nucleic acid	(16, 68)
1,044	C-C, C-O, C-N str, C-O-H	Peptides	(58)	1,545	υ(C = C) stretch., Tyr	Protein	(68)
1,054	Nucleic acids, CO str; Protein, C-N str	Nucleic acids, Protein	(67)	1,553	ν(OH)	Tryptophane	(69)
1,079	PO**_2_** str., (C–C) stretch., C–O	Nucleic acid, lipid, carbohydrates	(64)	1,573	Guanine, Adenine; Amide II	Guanine, Adenine; Amide II	(58)
1,085	C-O str	C-O str	(60)	1,575 to 1,578	Guanine, Adenine (ring str)	Guanine, Adenine	(60)
1,098	Phosphate, CC skeletal and COC str	Phosphate	(59)	1,582	Protein	Protein	(60)
1,099	CC skel, COC a-str, PO**_2_** str	CC skel, COC a-str, PO**_2_** str	(58)	1,589	ν(C − C)	Cyt c.	(20)
1,101	Symmetric phosphate stretch. (DNA)	Nucleic acid	(16)	1,593	Protein	Protein	(60)
1,102	>PO**_2_**- str (sym)	>PO**_2_**- str (sym)	(60)	1,599	υ(C = C) aromatic compound	Phenylalanine, Tyrosine	(16, 64)
1,123	CH Phe	Cytochrome	(16, 64)	1,604	Phenylalanine	Phenylalanine	(58)
1,127	=C-C= (unsaturated fatty acids in lipids)	Lipids	(59)	1,610	υ(C = C), Trp	Protein	(16, 64)
1,129	ν(C − N)	Cyt c.	(20, 60)	1,614	Tyrosine	Tyrosine	(60)
1,145 to 1,160	C-C, C-O ring breath, assym	C-C, C-O ring breath, assym	(70)	1,650 to 1,680	Amide I	Amide I	(60)
1,206	Aromatic Amino Acids	Aromatic Amino Acids	(69)	1,658	Unsaturated lipids	Lipids, Unsaturated fatty acid, protein	(61)
1,209	C–C**_6_**H**_5_** stretch., Phe, Trp	Protein	(64)	1,662	Amide I	Amide I	(59)
1,230 to 1,240	Amide III (α-helices)	Amide III (α-helices)	(63)	1,663	Amide I	Amide I	(60)
1,240	Thymine, Cytosine, Adenine, ring ν	Thymine, Cytosine, Adenine	(67)				

### Ability of Raman-DIP to quantify drought-tolerant cells in soil samples.

**FIG 5:**
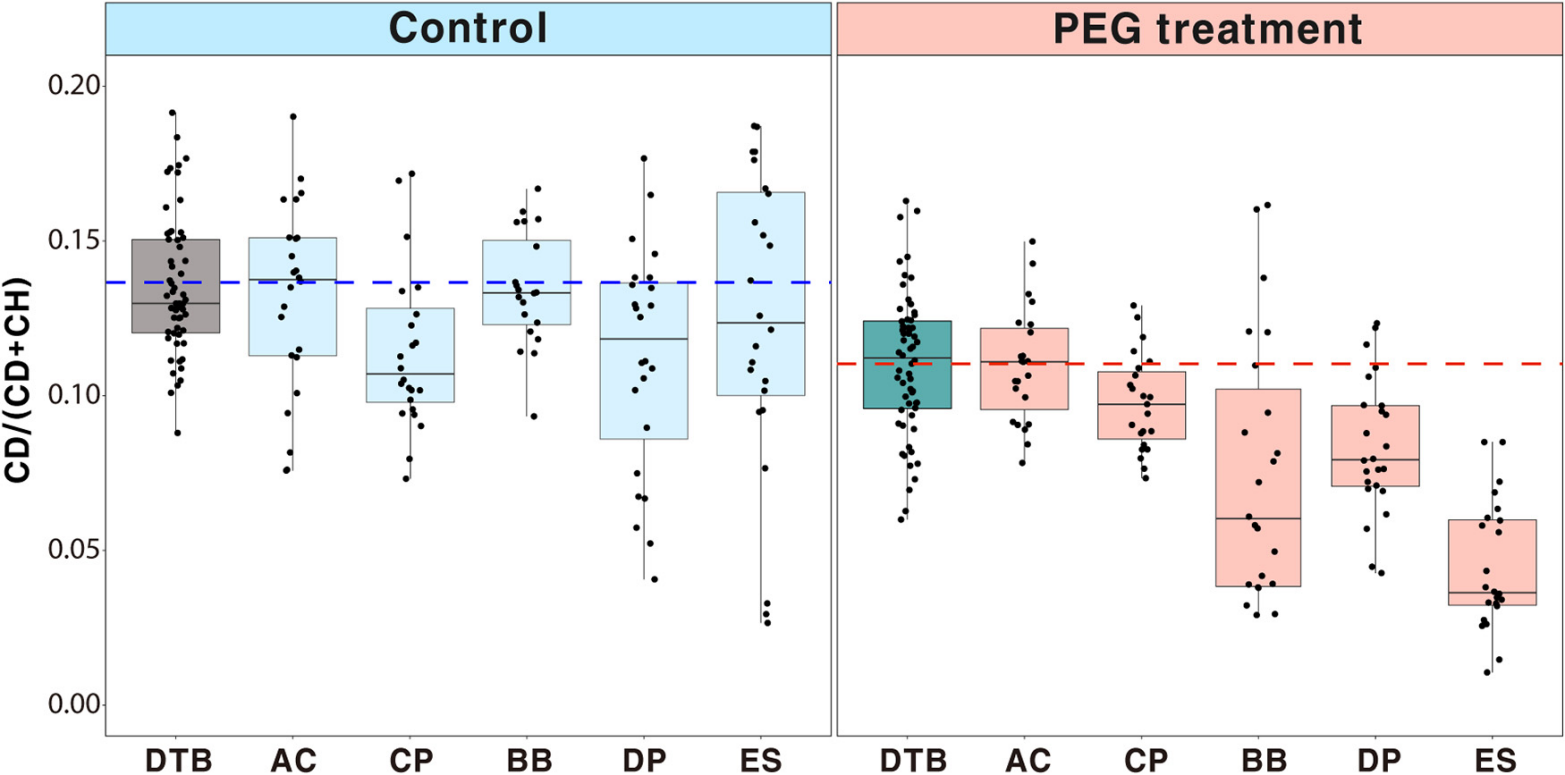
Evaluation of drought-tolerant capabilities of soil samples using C-D ratio. The C-D ratios are plotted as box plots. The mean of the C-D ratios of DTB in the control treatment is shown by the dashed blue line; that of DTB in the PEG treatment is shown by the dashed red line. DTB comprise the SCRS of three different model bacteria (*A. piechaudii*, *A. halopraeferens,* and *A. chlorophenolicus*).

Raman-DIP could identify the soil microorganisms with metabolic activity in both control and PEG treatments (Fig. S2 and 5). The C-D ratio of DTB in the control was significantly higher than that of the PEG treatment (*t* test, *P < *0.01), although both conditions showed high heterogeneity regarding the individual DTB cells (Fig. 5). Soil microorganisms generally had higher metabolic activity in the control (average: 0.12, range: 0.03 to 0.19) than in the PEG treatment (average: 0.08, range: 0.01 to 0.16), regardless of the type of soil sample (*t* test, *P < *0.01). Bacterial cells with high metabolic activity were defined as above-average cells with a DTB (C-D ratio of 0.14 in the control treatment and 0.11 in the PEG treatment). The high metabolic cell count obtained under PEG treatment was considered to represent drought-tolerant cells in the soil samples. Interestingly, the number of bacterial cells with the high metabolic activity did not differ from all samples in the control groups, whereas there was a significant difference in the number of drought-tolerant cells for each soil sample (ANOVA, *P < *0.01). Grown corn (AC, 52.2%) showed highest proportion of drought-tolerant cells, followed by peppers (CP, 21.7%), beans (BB, 21.7%), peppers (DP, 12.5%), and sweet potato (ES, 0%; Fig. S3). Correlations between soil properties and the proportions of drought-tolerant cells in each sample were analyzed to identify the soil properties that were correlated with drought tolerance; these correlations are summarized in Table S3. There were no significant relationships between the proportion of drought-tolerant cells and the soil physicochemical properties.

10.1128/msystems.01249-21.2 FIG S2 Average Raman spectra obtained from single cells after PEG treatment with D_2_O. SCRS were averaged (solid line), with SD shown in gray shading. DTB represent the SCRS of three drought-tolerant bacteria (*A. piechaudii, A. halopraeferens*, and *A. chlorophenolicus*). Download FIG S2, TIF file, 1.3 MB. Copyright © 2022 No et al. 2022 No et al. https://creativecommons.org/licenses/by/4.0/ This content is distributed under the terms of the Creative Commons Attribution 4.0 International license.

10.1128/msystems.01249-21.3 FIG S3 Proportions of drought-tolerant cells in soil samples after PEG treatment. Y-axis (Raman active cell) indicates the ratio of the number of bacterial cells with higher C-D ratios than the mean C-D ratio of DTB (0.11) to the total number of bacterial cells in each sample. Download FIG S3, TIF file, 0.3 MB. Copyright © 2022 No et al. 2022 No et al. https://creativecommons.org/licenses/by/4.0/ This content is distributed under the terms of the Creative Commons Attribution 4.0 International license.

10.1128/msystems.01249-21.8 TABLE S3 Correlations between soil properties and proportions of drought-tolerant bacterial cells. Download Table S3, DOCX file, 0.03 MB. Copyright © 2022 No et al. 2022 No et al. https://creativecommons.org/licenses/by/4.0/ This content is distributed under the terms of the Creative Commons Attribution 4.0 International license.

### Cross-validation of Raman-based phenotype by plant behavior under drought stress.

Plant cultivation experiments, which compared the number of revived plants after re-watering after drought stress to the plants in each soil sample, were conducted to assess whether the soil microbiome improved PGP under drought conditions. The association between the drought tolerance of the soil microbiome and PGP was investigated by comparing the results of the plant cultivation experiments with the proportions of drought-tolerant cells determined via Raman-DIP. The proportions of revived plants under drought were highest for AC (40%) and CP (40%), followed by BB (20%; Fig. S4A); no revived plants were observed in DP or ES. These results showed significantly similar trends to the quantitative drought tolerance analysis of the soil microbiome, in which AC and CP ranked highly, whereas DP and ES were both low (rho = 0.9, *P* < 0.05) (Fig. S4B).

10.1128/msystems.01249-21.4 FIG S4 Evaluation of alleviation capacity of soil microbial community through pot tests. Sixteen replicate plants were grown for each soil (A). Correlation between the proportion of Raman active cell and the proportion of revived plants in each soil sample under drought conditions (rho = 0.9, *P* < 0.05) (B). Download FIG S4, TIF file, 0.5 MB. Copyright © 2022 No et al. 2022 No et al. https://creativecommons.org/licenses/by/4.0/ This content is distributed under the terms of the Creative Commons Attribution 4.0 International license.

### Metagenomic analysis of the soil samples.

**FIG 6:**
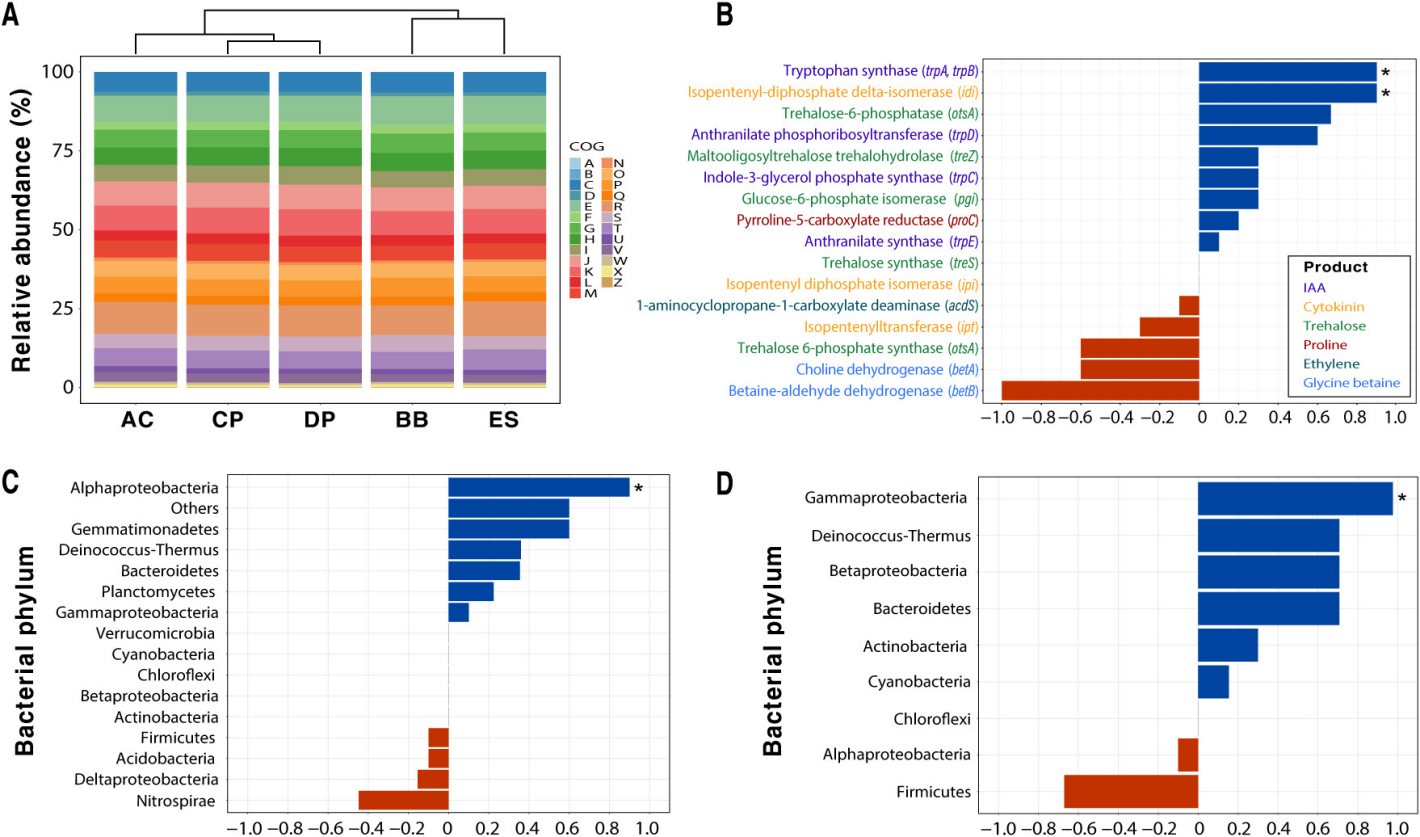
Functional classification and correlation with proportion of drought-tolerant cells. (A) Functional classification based on COG for the microbiome in each soil; upper plot shows a similarity tree. (B) Correlations between abundances of functional products involved in important metabolic pathways for drought tolerance and proportions of drought-tolerant cells in soil samples. Correlations between relative abundances of the bacteria producing (C) tryptophan synthase or (D) isopentenyl-diphosphate delta-isomerase, shown alongside proportions of drought-tolerant cells in soil samples (*, *P* < 0.05).

Shotgun metagenomic approaches revealed the genotypic information of the soil microbiome regarding drought tolerance and PGP, as summarized in Table S4. In all soil samples, the predominant phyla were *Actinobacteria* (37.0% to 51.0%), *Alphaproteobacteria* (13.3% to 30.4%), *Gammaproteobacteria* (3.2% to 12.0%), and *Betaproteobacteria* (3.4% to 7.0%; Fig. S5). Cluster analysis revealed that the taxonomic profiles of the microbial communities were divided into two groups (Fig. S5). AC and CP, which showed high proportions of drought-tolerant cells in the soil microbiome, were taxonomically most similar to ES and DP, which showed the lowest proportions. However, COG-based clustering analysis revealed that AC was functionally similar to CP, although CP and DP were similar from both taxonomic and functional perspectives (Fig. 6A). The macroscopic results of genotypes from these metagenomic approaches did not match perfectly with the spectral and plant phenotypic results.

10.1128/msystems.01249-21.5 FIG S5 Cluster tree bacterial classifications at the phylum level for the five soil samples. Download FIG S5, TIF file, 0.6 MB. Copyright © 2022 No et al. 2022 No et al. https://creativecommons.org/licenses/by/4.0/ This content is distributed under the terms of the Creative Commons Attribution 4.0 International license.

10.1128/msystems.01249-21.9 TABLE S4 Comparison of assemblies from reads of different soil samples. Download Table S4, DOCX file, 0.03 MB. Copyright © 2022 No et al. 2022 No et al. https://creativecommons.org/licenses/by/4.0/ This content is distributed under the terms of the Creative Commons Attribution 4.0 International license.

The associations between genotypes and phenotypes were further analyzed by selecting microbial gene products that improved plant growth under drought, such as the biosynthesis of osmolytes and the production of phytohormones (26–28). Regarding the correlations between the abundance of gene products and the proportions of drought-tolerant cells in the soil microbiome, tryptophan synthase (rho = 0.900) and isopentenyl-diphosphate delta-isomerase (rho = 0.900), which are both related to phytohormone production, were positively and significantly associated with the proportion of DTB (*P < *0.05; Fig. 6B). Trehalose-6-phosphatase, which acts as a homeostatic regulator of osmotic levels, was also relatively highly correlated with the proportion of drought-tolerant cells in the soil microbiome. Unexpectedly, betaine aldehyde dehydrogenase and choline dehydrogenase, which catalyze the synthesis of glycine betaine and choline as regulators of osmosis, respectively, were negatively correlated with the phenotypic results. Tryptophan synthase and isopentenyl-diphosphate delta-isomerase were highly associated with the abundance of *Alphaproteobacteria* (rho = 0.900) and *Gammaproteobacteria* (rho = 0.975), respectively (Fig. 6C and D).

## DISCUSSION

Raman spectroscopy was able to measure bacterial drought tolerance through changes in phenotypes and isotope markers under drought stress. There were no significant changes in the SCRS of DTB with or without drought stress. If bacterial phenotypes under normal conditions remained unchanged under stress conditions, this implies that bacteria are tolerant to the stress. This finding is consistent with those of other phenotype studies of bacterial cells regarding abiotic stress resistance using Raman spectroscopy (23–25). A previous study reported that the heterogeneity of SCRS in resistant Escherichia coli strains were relatively lower than those of sensitive strains when treated with different antibiotics at higher-than-MICs (17). Here, the present study found a “drought effect signature” that was expressed by specific Raman peaks in DSB; this signature showed changes in SCRS, with reduced lipids and increased proteins. Such changes in the biomolecules of DSB are typical phenotypes when bacterial cells are damaged by osmotic pressure (29). The observed increase in the protein to lipid ratio is also an important Raman phenotype indicator in which cells progress into death phases under stress (18). Taken together, Raman spectroscopy could provide an unequivocal discrimination for identifying phenotypic differences between DTB and DSB when under drought stress.

The results of this study demonstrate that Raman-DIP can be used to assess drought-tolerant bacterial cells in soil samples by utilizing isotopic spectral information. Here, the SCRS of DTB had a detectable C-D band under both control and PEG conditions, whereas the C-D band in DSB disappeared under drought conditions. The presence of C-D bands in SCRS under drought conditions can thus indicates metabolic activity regarding drought tolerance. D_2_O labeling methods can be used as a universal labeling technique, without additional isotope labeled substrates, to identify metabolically active cells in a given environment. This allows for the observation of the C-D band in metabolically active cells (30). Thus, the C-D band in SCRS can be used as a major indicator of metabolic activity against abiotic stresses such as antibiotics, carbon starvation and UV dosage (23–25). In addition, C-D bands clearly vary in intensity according to the dose-response effect, so its intensity varies proportionally with the strength of the stress that the bacterial cells in question are reacting to. A previous study found that increasing the UV dosages from 10 to 200 mJ/cm^2^ proportionally reduced the C-D ratios of tested strains from 95.7% to 47.9% (compared with controls), depending on the level of tolerance to the UV dose (25). The similar intensity of the DTB C-D band in both the control and PEG treatments indicates that DTB maintain high metabolic activity even in the given extreme drought environment.

This study evaluated the drought tolerance of the soil microbiome by treating with 20% PEG, which was 5% lower than for an axenic culture. The drought-tolerant capability of DTB in an axenic culture can be overestimated due to rapid metabolism in response to stress adaptation or a reduced nutrient availability (31). In addition, most soil bacteria remain dormant or have low activities due to low nutrient availability, and are sensitive to external stresses (32). Even considering the heterogeneity of metabolic activity in an individual cell, the proportions of drought-tolerant cells with high metabolic activity were remarkably lower in soil samples compared with those in DTB in an axenic culture. The proportions of drought-tolerant cells in each soil sample differed significantly, indicating that Raman-DIP can be a useful approach for comparing the drought-tolerant capability of the microbiome for different soil types.

A close connection was observed between the phenotypes of plants and the soil microbiome by examining plant cultivation under drought conditions. The proportions of revived plants under drought and the proportions of drought-tolerant cells in each soil were very similar. Of the soils tested, both AC and CP had the highest proportions of revived plants and drought-tolerant cells, whereas CP and DP had no revived plants and had the lowest rates of drought tolerant cells. These findings suggest that the proportion of drought-tolerant cells in a soil microbiome is closely related to PGP under drought. The soil samples collected in this study came from an area with a long history of cultivating various crops; thus, they were likely to have already been strongly selected for symbiotic microorganisms with crops. Jarvis et al. examined the deterministic process of the rhizosphere microbiome by investigating agricultural management and crop types (33). In this respect, bacteria that are identified as being symbiotic with crops through deterministic processes likely harbor the best potential as a drought-tolerant cells that can be detected by Raman-DIP.

An integrative investigation of drought tolerance in soils is required to improve the current understanding of the reliable link between Raman-based phenotype and metagenomics-based genotype analyses. The gene abundances of tryptophan synthase and isopentenyl-diphosphate delta-isomerase related phytohormones were found to be positively associated with the proportion of drought-tolerant cells in this study. IAA and cytokinin are produced by symbiotic bacteria such as *Azospirillum* sp., Rhizobium leguminosarum, and Bacillus subtilis; they promote plant root development by increasing the uptake of nutrients and water under drought conditions (34–36). The results presented here support the existence of a strong association between DTB abundance and the abundance of soil bacteria that are able to promote plant growth in drought conditions, assuming that the phenotype and genotype of the soil microbiome are consistent. Simultaneously tracking associations regarding the phenotypic and genotypic features of the microbiome offers opportunities to address gaps in the current understanding of microbial responses to environmental stress. It is noteworthy that one drought tolerance strategy, osmolyte biosynthesis, was found to be primarily negatively correlated with DTB abundance. Osmolytes, including glycine, betaine, and choline, are known to temporarily accumulate in cytoplasm in the early stages of bacterial cells under stress conditions, but have been shown to disappear sharply as the cells enters the second half of the exponential phase (37). The abundance of osmolyte-producing bacteria can be overlooked when estimating the abundance of DTB due to the decreasing activity of osmolyte-producing bacteria with long incubation periods, and due to the increasing number of bacteria that externally acquire osmolytes. As the DTB studied here may result from bacteria that adapted to drought conditions for 48 h and produced plant hormones, further studies are needed to study how metabolically active strains vary depending on the duration of drought stress.

Additionally, by analyzing the correlation between the taxonomic information and gene products of plant hormones obtained from metagenomic approaches, this study examined which phyla can promote plant growth in drought conditions. *Alphaproteobacteria* and *Gammaproteobacteria*, which have genes related to the production of tryptophan synthase and isopentenyl-diphosphate delta-isomerase, were significantly positively correlated with the proportion of drought-tolerant cells. *Azospirillum*, *Sphingomonas*, Pseudomonas, and Enterobacter, belonging to two phyla, are known to be symbiotic microorganisms that promote plant growth by producing plant hormones in drought conditions; these relationships have been identified through incubation or transcriptomic approaches (26, 34, 38, 39). Although a plant’s genotype makes up a dependent symbiotic microbiome (the crops grown in each soil type varied in this study), bacteria that reduce plant stress in drought conditions are likely to belong to these phyla (40). Further investigation of these results regarding the detailed genomic features seems necessary; this could be achieved by sorting bacteria with high metabolic activity against drought resistance using a Raman-activated cell sorting system, and by performing single cell genomics (41).

In summary, this study demonstrated that Raman-DIP, aided with metagenomics-based approaches, can establish a reliable link between the phenotype and the genotype in the soil microbiome regarding drought tolerance, and regarding PGP under drought conditions. These findings provide a fundamental insight into the association between drought tolerance and the soil microbiome, and pave the way for comprehensive assessment tools regarding the drought tolerance of the soil microbiome. These assessment tools could be linked to technologies that can assess soil microbiome resistance or adaptation to disturbances driven by climate change, or by pollution arising from human activities.

## MATERIALS AND METHODS

### Strains and cultivation conditions.

Six bacterial strains were selected, including the DTB A. piechaudii (KACC 20750), Arthrobacter chlorophenolicus Au4 (KACC 13166), and A. halopraeferens (ATCC 43552) (42–44), and the DSB Azospirillum lipoferum VPI Sp 59b (KACC 13157), Derxia gummosa (ATCC 15994), and R. soli DS-42 (KCTC 12873) (44, 45). *A. piechaudii*, *A. lipoferum*, *D. gummosa*, and *R. soli* were obtained from Korean Collection of Type Cultures (KCTC). *A. chlorophenolicus* and *A. halopraeferens* were obtained from the Korean Agricultural Culture Collection (KACC, Korea). The media and incubation conditions are summarized in Table S1.

10.1128/msystems.01249-21.6 TABLE S1 Culture media and incubation conditions for microorganisms. Download Table S1, DOCX file, 0.03 MB. Copyright © 2022 No et al. 2022 No et al. https://creativecommons.org/licenses/by/4.0/ This content is distributed under the terms of the Creative Commons Attribution 4.0 International license.

PEG, which is a high-molecular-weight osmotic substance solution, controls the osmotic pressure in bacterial cells. Thus, it can be used as an artificial drought stressor (46). The bacterial cells were inoculated with OD600 0.1 of exponentially grown bacterial cultures in 20 ml of the media with 25% PEG 6,000 (Sigma-Aldrich, Missouri, United states), which was used to simulate drought conditions (−1.25 MPa) for 24 h (47). The bacterial cells extracted from the soil samples were incubated in 20 mL of nutrient broth including 20% (−1.09 MPa) PEG 6,000 at 5°C, while being shaking at 120 rpm for 48 h (48). Samples without PEG treatment were used as a control. D_2_O labeling was performed using a modified method based on that employed in a previous study (22). Forty percent of the distilled water in the media was replaced with D_2_O water.

### Soil sampling and physicochemical analysis.

Soil samples were collected at a depth of 10 cm from agricultural land in Wonju, Gangwon-do, South Korea. The soil samples were collected from arable soil being used to grow corn (AC), beans (BB), peppers (CP and DP), and sweet potato (ES). Information regarding sampling sites and physicochemical properties is summarized in Table S2 and Text S1. The samples were sieved through a 2 mm sieve to homogenize them, and were then stored at −80°C prior to analysis.

10.1128/msystems.01249-21.7 TABLE S2 Soil sample collection sites and physicochemical properties of soil samples. Download Table S2, DOCX file, 0.03 MB. Copyright © 2022 No et al. 2022 No et al. https://creativecommons.org/licenses/by/4.0/ This content is distributed under the terms of the Creative Commons Attribution 4.0 International license.

10.1128/msystems.01249-21.10 TEXT S1 Physicochemical analysis. To determine the pH, 2 g of each air-dried soil sample was added into 10 mL of deionized water, vortexed and left to stabilize for 10 min prior to measurement. The pH of a 1:5 soil:water solution was measured with a pH meter (Orion Star A211, Thermo Scientific, USA) (71). To determine the gravimetric soil moisture, the field-wet soil mass was measured and the soil was then dried at 105°C for 48 h. Soil moisture was calculated using the difference between field moist mass and oven dried mass [(wet mass − dry mass)/(dry mass) × 100] (72). Soil texture was measured using the standard determination by gravitational sedimentation method (73). The total nitrogen (TN) was measured using the Kjeldahl digestion procedure (74). The total phosphorus (TP), in the form of PO_4_^−^, was determined using the vanadomolybdophosphoric acid colorimetric method (72). The dissolved organic carbon (DOC) and total organic carbon (TOC) levels were determined using a TOC Analyser (Shimadzu, Japan). Download Text S1, DOCX file, 0.03 MB. Copyright © 2022 No et al. 2022 No et al. https://creativecommons.org/licenses/by/4.0/ This content is distributed under the terms of the Creative Commons Attribution 4.0 International license.

### Collection of bacterial cells from soil samples.

Bacterial cells were extracted from soil samples using Nycodenz density gradient separation. First, 2 g of each soil sample was measured into a 15-mL sterile conical tube and 10 mL of 1X phosphate buffer saline (PBS: NaCl 8 g L^−1^, KCl 0.2 g L^−1^, Na_2_HPO_4_ 1.44 g L^−1^, KH_2_PO_4_ 0.24 g L^−1^, pH 7.4) buffer was added and mixed with 0.5% (vol/vol) Tween 20, 0.35% wt/v polyvinylpyrrolidone and 3 mM sodium pyrophosphate. After being vortexed for 30 min, 1 vol of the slurry was added to 1 vol of Nycodenz (1.42 g mL^−1^, Sigma-Aldrich, Missouri, United States) and centrifuged at 14,000 rpm for 90 min at 4°C. The middle and upper aqueous layers containing bacterial cells were carefully collected in a new centrifuge tube. The bacterial cells were then collected by centrifuging them at 9,000 rpm for 10 min and washing the remaining solution with ultrapure water (49). The bacterial cells were washed in PBS buffer by centrifuging them at 13,000 rpm for 5 min. The samples were then fixed with 4% formaldehyde at 4°C for 2 h and then washed twice with PBS buffer.

### Raman spectra acquisition.

The samples were prepared for Raman microspectroscopy analysis by spotting 1.5 μL of each fixed bacterial cell sample onto an aluminum coated slide (LiMedion, Mannheim, Germany), which was then air dried at 23°C room temperature. Each slide was washed with ultrapure water to remove the saline and then air dried. The Raman spectra were acquired using a Confocal Raman imaging system XperRam35V (Nanobase, Seoul, South Korea) equipped with 1,800 g/mm grating, a 532 nm neodymium-yttrium aluminum garnet laser, LTGL-532RL (Leading tech, Shanghai, China), and a MPLFLN 40X objective (Olympus, Tokyo, Japan). The laser power on a single cell was 2.0 mW. The acquisition time of each spectrum was 25 s for each single cell. The resulting scattered light was collected on an Atik 428EX Color charged-coupled device (CCD) Camera (Atik cameras, Bawburgh, UK) that was cooled at −70°C.

### Raman spectral processing.

Raman spectra were collected from 20 individual cells for all tested conditions. All data processes of Raman spectra were done with the R package “Chemospec” (50). The spectra were pretreated using the function “baselineSpectra” with method “als” for baseline correction by second derivative constrained weighted regression. The function “normSpectra” was used with method “TotInt” for the normalization, which a way to normalize the spectra through dividing the total intensity by the relative intensity. The function “clupaSpectra” was used for peak alignment with default value. Raman shifts ranging from 400 to 1,800 cm^−1^ were used to analyze the bacterial phenotypes (51).

The peak areas assigned to C-D (2,040 to 2,300 cm^−1^), C-H (2,800 to 3,100 cm^−1^), and the silence region (2,400 to 2,700 cm^−1^) were integrated using the function “integrate” in the R package “stats.” The ratio of (C-D – silence region)/[(C-D – silence region) + (C-H – silence region)] (C-D ratios) was calculated to quantify the level of deuterium incorporation in 20 to 25 bacterial cells of each soil sample. The silence region was adopted to decrease the effects of noise for the Raman spectra. The ratio of proteins to lipids was calculated by dividing the integrated area of 1,209 cm^−1^ by the area of 1,267 cm^−1^ (18).

### Plant cultivation test.

The plant growth promotion capacity of the soil microbiome under drought conditions was evaluated using a pot test. The surface of pea (Pisum sativum L.) seeds were sterilized with 2.5% NaOCl for 3 min and then washing five times with running deionized water. The seeds were kept in a growth chamber for 3 days for pregermination with moisture. Then, 15 g of soil was placed in each pot and one pregerminated seed was sown per pot. During the first 2 weeks, pots were fully watered on alternating days, following which drought stress was applied over the next 7 days by restricting watering. After 7 days of drought stress, plants were rewatered for 3 days and then harvested. The pot test was conducted in a growth chamber with 30/14°C (day/night) temperatures, 14/10 h (day/night) periods, and 50% relative humidity. At the end of the experiment, the numbers of revived green leaves were counted. The experiment was repeated twice, with 10 replicates for each soil sample. We expressed as a percentage the total number of counted plants in two replicates.

### Metagenome sequencing.

Total genomic DNA was extracted from soil samples after 1 week of drought stress using the FastDNA™ Spin Kit for Soil (MP Biomedicals, Irvine, USA). DNA quality was measured using a Quanti-iT PicoGreen dsDNA assay kit (Invitrogen, Waltham, USA). Library construction and sequencing was conducted with an Illumina HiSeq Xten platform (Illumina, San Diego, USA) by Microgen (Seoul, South Korea). The Illumina adapters were removed using BBTools v.38.22 with default parameters in KBase (52). The sequence data were quality filtered using BBTools to remove low quality reads and correct the sequencing errors with default parameters (52). Filtered reads were assembled using MEGAHIT v1.2.9 using a minimum threshold of 300 bp (bp) contigs. The integrated microbial genomes with microbiome samples (IMG/M) annotation pipelines were used to annotate the metagenomic sequence data (53). All genomes were annotated to predict their bacterial classifications with protein-coding genes using Prodigal v.2.6.3. Functional gene annotation was performed using the IMG/M via Cluster of Orthologous Genes and Kyoto Encyclopedia of Genes and Genomes Orthology analyses (53).

### Statistical analysis.

All statistical analyses were performed using R (v. 3.6.3) (54). Discriminant analysis of principal components (DAPC) was conducted for all processed spectra to identify Raman spectra signatures of DTB. The DAPC was conducted by employing the principal-component analysis scores of as explanatory variables of the discriminant analysis using the R package “adegenet” (55). The distances in the DAPC plots were calculated and the average and standard deviation were calculated between each centroid of each control and each dot of each PEG-treated group. To calculate the significance of differences between control and PEG treated samples in the DAPC which was expressed as LD1 and LD2, we conducted the Wilcoxon test for LD1 and LD2 of control and PEG treated samples in each species.

To calculate the significance of differences in the SCRS, Wilcoxon tests or *t*-tests were performed based on the results of a normality test which was conducted using the Shapiro test. Dendrograms were constructed at the phylum level using hierarchical clustering based on the Bray-Curtis Dissimilarity index, using the R package “vegan” (56). Spearman rank correlations between the proportion of drought-tolerant cells and the abundance of gene products were calculated using the R function “cor.test.”

### Data availability.

The metagenome sequences were deposited in the IMG/M web site (https://img.jgi.doe.gov) under the accession number Ga0477069, Ga0483887, Ga0483885, Ga0483897, and Ga0477070.
