# Supplementary figures and images for: Raman-Deuterium Isotope Probing and Metagenomics Reveal the Drought Tolerance of the Soil Microbiome and Its Promotion of Plant Growth
Source: mSystems. 2022 Feb 1;7(1):e01249-21. doi: 10.1128/msystems.01249-21 (PMC8805637; doi:10.1128/msystems.01249-21)

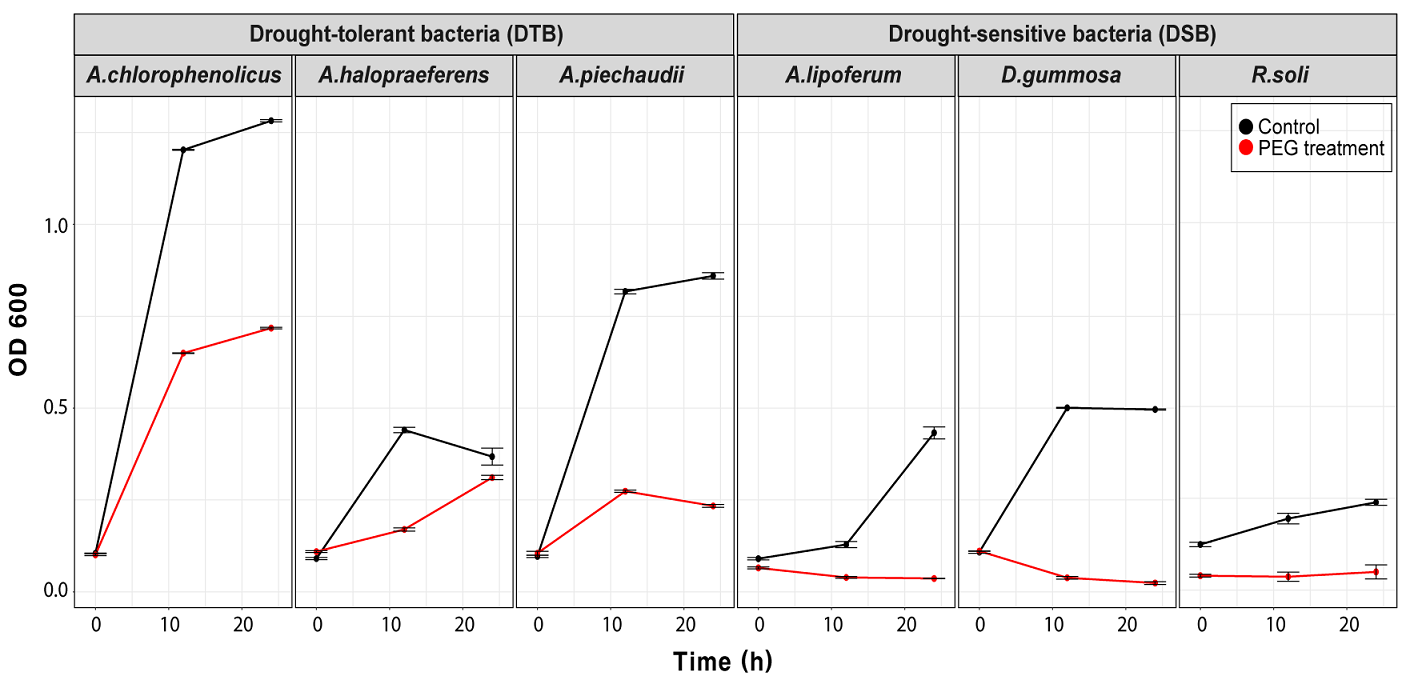

Supplement: FIG S1 [file msystems.01249-21-sf001.tif]

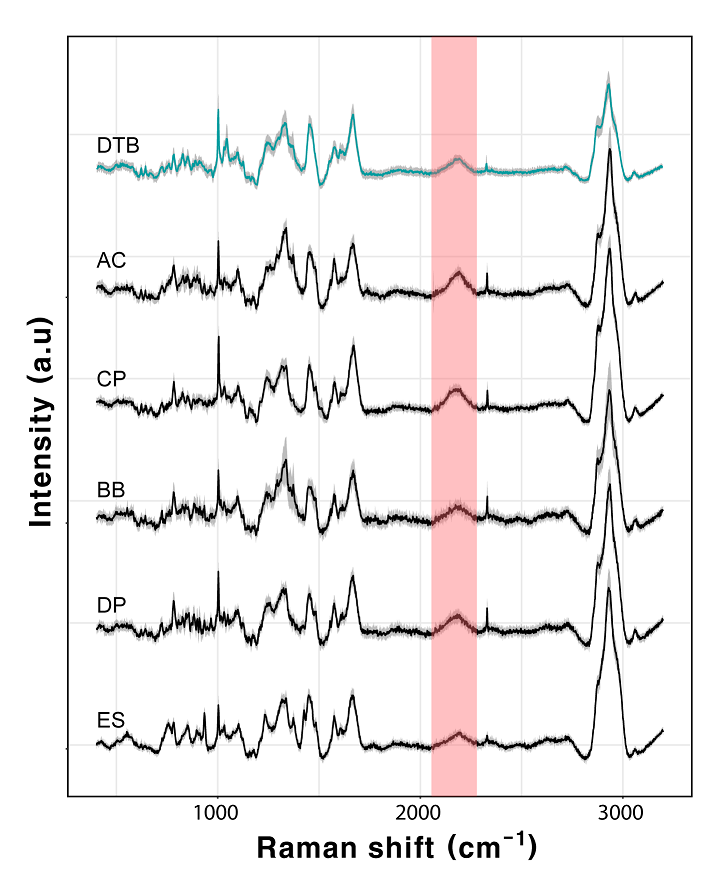

Supplement: FIG S2 [file msystems.01249-21-sf002.tif]

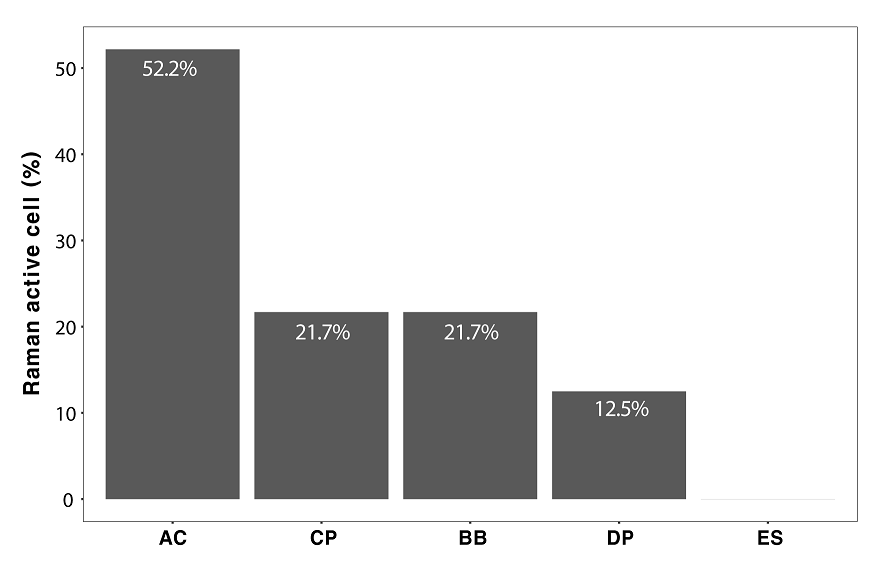

Supplement: FIG S3 [file msystems.01249-21-sf003.tif]

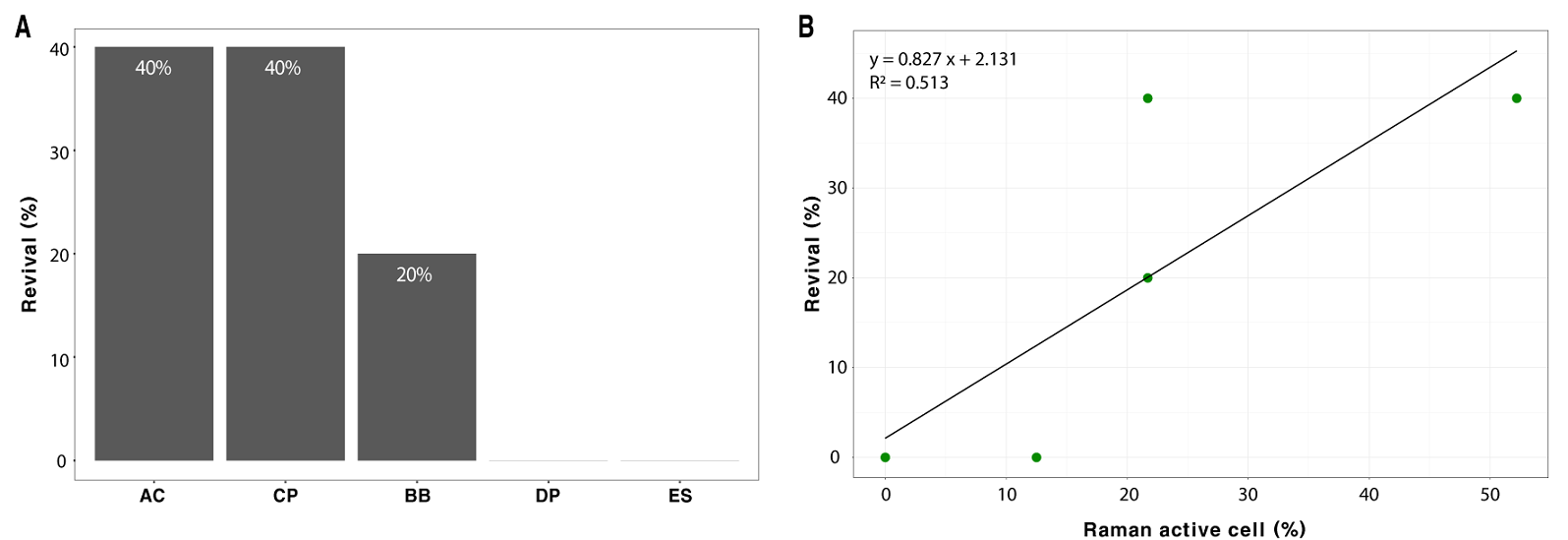

Supplement: FIG S4 [file msystems.01249-21-sf004.tif]

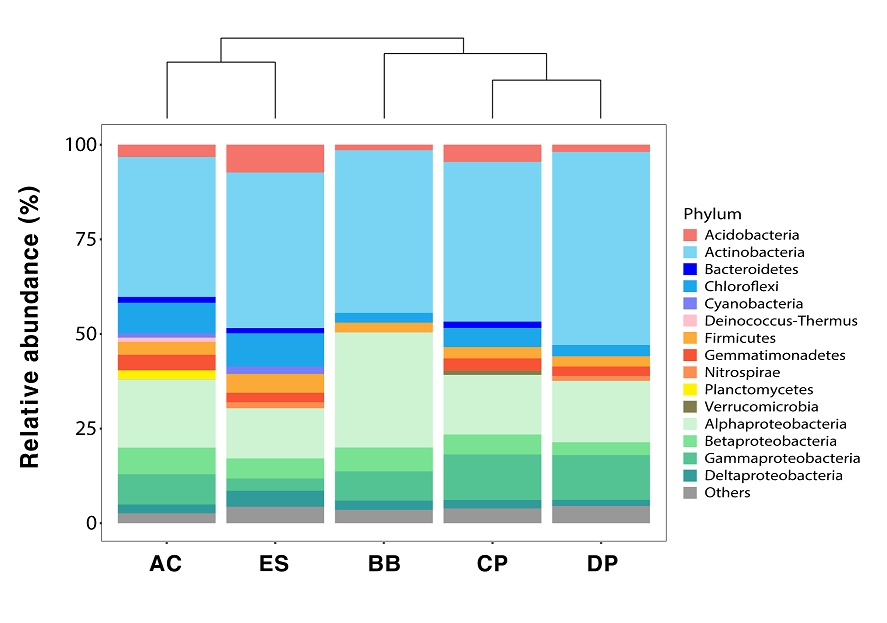

Supplement: FIG S5 [file msystems.01249-21-sf005.tif]
