# Supplementary material for: Raman-Deuterium Isotope Probing and Metagenomics Reveal the Drought Tolerance of the Soil Microbiome and Its Promotion of Plant Growth
Source: mSystems. 2022 Feb 1;7(1):e01249-21. doi: 10.1128/msystems.01249-21 (PMC8805637; doi:10.1128/msystems.01249-21)
Supplement: TABLE S3 [file msystems.01249-21-st003.docx]

**Table S3. Correlations between soil properties and proportions of drought-tolerant bacterial cells.**

| Soil properties | Cor (rho) | p-value |
| --- | --- | --- |
| pH | -0.368 | 0.5418 |
| Moisture | 0.051 | 0.9347 |
| Clay | -0.026 | 0.9665 |
| Silt | 0.658 | 0.2275 |
| Sand | -0.462 | 0.4338 |
| TN | -0.462 | 0.4338 |
| TP | 0.103 | 0.8696 |
| TOC | -0.872 | 0.0539 |
| DOC | -0.359 | 0.5528 |
