# Supplementary material for: Raman-Deuterium Isotope Probing and Metagenomics Reveal the Drought Tolerance of the Soil Microbiome and Its Promotion of Plant Growth
Source: mSystems. 2022 Feb 1;7(1):e01249-21. doi: 10.1128/msystems.01249-21 (PMC8805637; doi:10.1128/msystems.01249-21)
Supplement: TABLE S4 [file msystems.01249-21-st004.docx]

**Table S4. Comparison of assemblies from reads of different soil samples.**

| **Samples** | **Total seqs** | **Quality filtered seqs** | **Assembled contigs (Largest contig [bp])** | **Annotated contigs** | **Assigned KO** | **Assigned COG** |
| --- | --- | --- | --- | --- | --- | --- |
| AC | 171,130,694 | 170,676,928 | 125,510 (165,031) | 120,503 | 99,799 | 119,856 |
| CP | 134,322,624 | 133,988,206 | 94,112 (119,515) | 94,109 | 76,139 | 89,994 |
| BB | 137,782,756 | 137,572,080 | 11,243 (31,061) | 11,243 | 9,120 | 10,910 |
| DP | 152,540,444 | 152,299,624 | 87,684 (310,777) | 87,684 | 72,276 | 84,919 |
| ES | 166,581,358 | 166,077,092 | 62,511 (153,126) | 62,511 | 47,610 | 59,693 |
