# Supplementary material for: Raman-Deuterium Isotope Probing and Metagenomics Reveal the Drought Tolerance of the Soil Microbiome and Its Promotion of Plant Growth
Source: mSystems. 2022 Feb 1;7(1):e01249-21. doi: 10.1128/msystems.01249-21 (PMC8805637; doi:10.1128/msystems.01249-21)
Supplement: TABLE S1 [file msystems.01249-21-st001.docx]

**Table S1. Culture media and incubation conditions for microorganisms.**

| **Drought** | **Microorganisms** | **Medium** | **Temperature (℃)** | **RPM** | **Incubation time (h)** |
| --- | --- | --- | --- | --- | --- |
| Tolerant | *Arthrobacter chlorophenolicus* | Tryptone soy broth | 30 | 120 | 24 |
| Tolerant | *Azospirillum halopraeferens* | Nutrient broth | 41 | 120 | 24 |
| Tolerant | *Achromobacter piechaudii* | Nutrient broth | 30 | 120 | 24 |
| Sensitive | *Azospirillum lipoferum* | Nutrient broth | 30 | 120 | 24 |
| Sensitive | *Derxia gummosa* | Nutrient broth | 30 | 120 | 24 |
| Sensitive | *Rhizobium soli* | Reasoner's 2A broth | 30 | 120 | 24 |
