# Supplementary material for: Raman-Deuterium Isotope Probing and Metagenomics Reveal the Drought Tolerance of the Soil Microbiome and Its Promotion of Plant Growth
Source: mSystems. 2022 Feb 1;7(1):e01249-21. doi: 10.1128/msystems.01249-21 (PMC8805637; doi:10.1128/msystems.01249-21)
Supplement: TABLE S2 [file msystems.01249-21-st002.docx]

**Table S2. Soil sample collection sites and physicochemical properties of soil samples.**

| **Samples** | **Sampling site** | **Crops** | **pH** | **Moisture**  **(%)** | **Soil texture (%)** | | | **TN**  **(mg/L)** | **TP**  **(mg/L)** | **TOC**  **(mg/L)** | **DOC**  **(mg/L)** |
| --- | --- | --- | --- | --- | --- | --- | --- | --- | --- | --- | --- |
|  |  |  |  |  | **Clay** | **Silt** | **Sand** |  |  |  |  |
| AC | 37°27′71.23″N, 127°90′30.91″E | Corn | 6.5 | 16.4 | 10.7 | 5.7 | 83.6 | 3.6±0.1 | 9.7±0.4 | 43.8 | 30.6 |
| BB | 37°29′18.45″N, 127°91′58.91″E | Bean | 7.5 | 17.4 | 9.8 | 7.7 | 82.5 | 13.9±0.1 | 1.8±0.4 | 61.3 | 37.5 |
| CP | 37°27′11.20″N, 127°90′43.01″E | Pepper | 6.1 | 17.1 | 11.9 | 5.7 | 87.4 | 5.8±0.2 | 5.2±2.8 | 58.9 | 37.7 |
| DP | 37°30′44.28″N, 127°91′11.18″E | Pepper | 6.7 | 22.5 | 9.8 | 2.4 | 87.8 | 10.1±0.0 | 12.2±0.2 | 74.6 | 48.2 |
| ES | 37°28′44.30″N, 127°91′19.50″E | Sweet potato | 6.7 | 13.7 | 11.4 | 3.2 | 85.4 | 6.2±0.2 | 4.7±0.3 | 63.4 | 35.1 |
