# Supplementary material for: Raman-Deuterium Isotope Probing and Metagenomics Reveal the Drought Tolerance of the Soil Microbiome and Its Promotion of Plant Growth
Source: mSystems. 2022 Feb 1;7(1):e01249-21. doi: 10.1128/msystems.01249-21 (PMC8805637; doi:10.1128/msystems.01249-21)
Supplement: TEXT S1 [file msystems.01249-21-s0001.docx]

# Supplemental Methods

**Text S1. Physicochemical analysis**

To determine the pH, 2 g of each air-dried soil sample was added into 10 mL of deionized water, vortexed and left to stabilize for 10 min prior to measurement. The pH of a 1:5 soil:water solution was measured with a pH meter (Orion Star A211, Thermo Scientific, USA) (71). To determine the gravimetric soil moisture, the field-wet soil mass was measured and the soil was then dried at 105 °C for 48 h. Soil moisture was calculated using the difference between field moist mass and oven dried mass [(wet mass − dry mass) / (dry mass) × 100] (72). Soil texture was measured using the standard determination by gravitational sedimentation method (73). The total nitrogen (TN) was measured using the Kjeldahl digestion procedure (74). The total phosphorus (TP), in the form of PO_4_^−^, was determined using the vanadomolybdophosphoric acid colorimetric method (72). The dissolved organic carbon (DOC) and total organic carbon (TOC) levels were determined using a TOC Analyser (Shimadzu, Japan).
